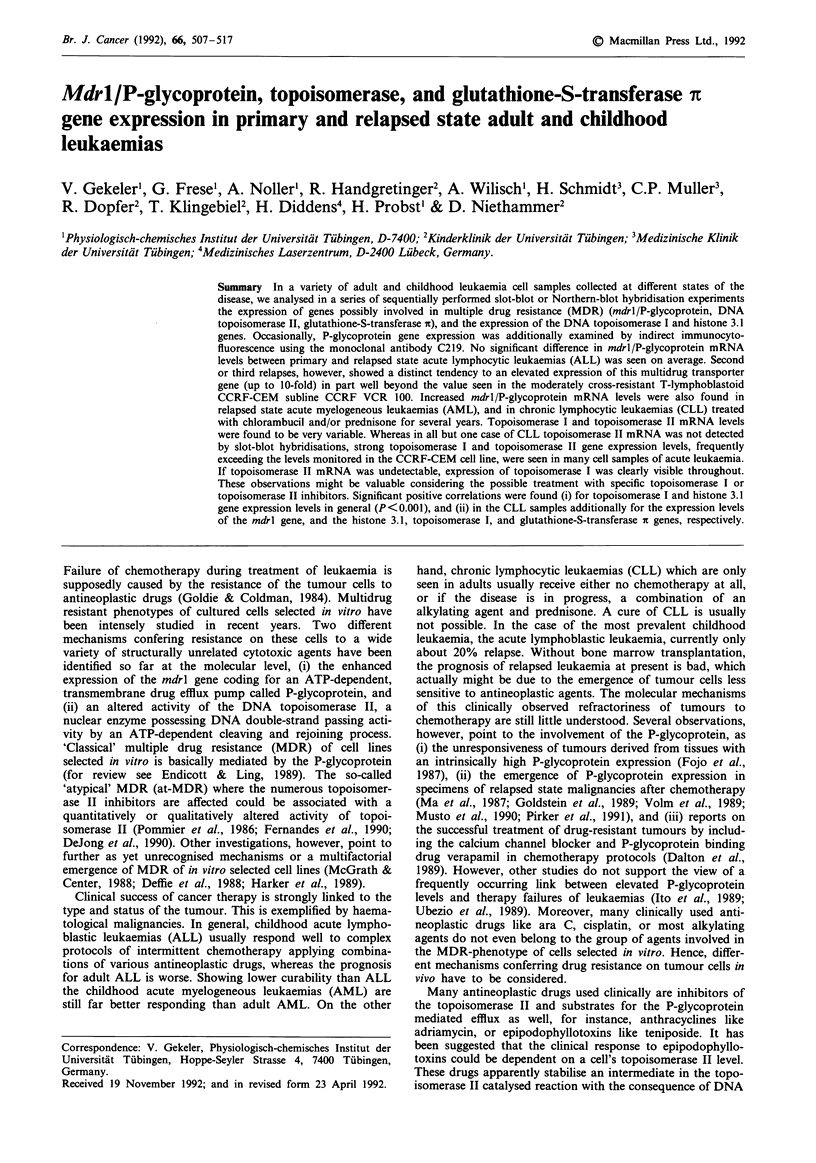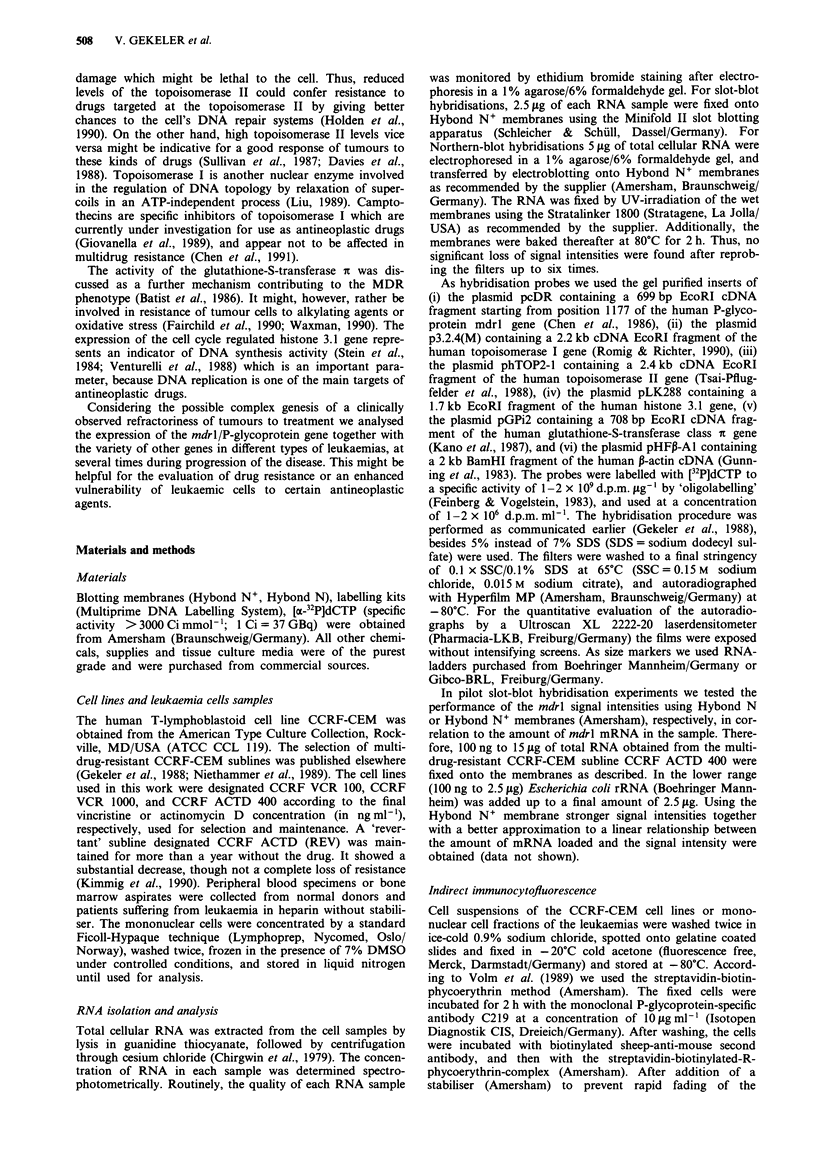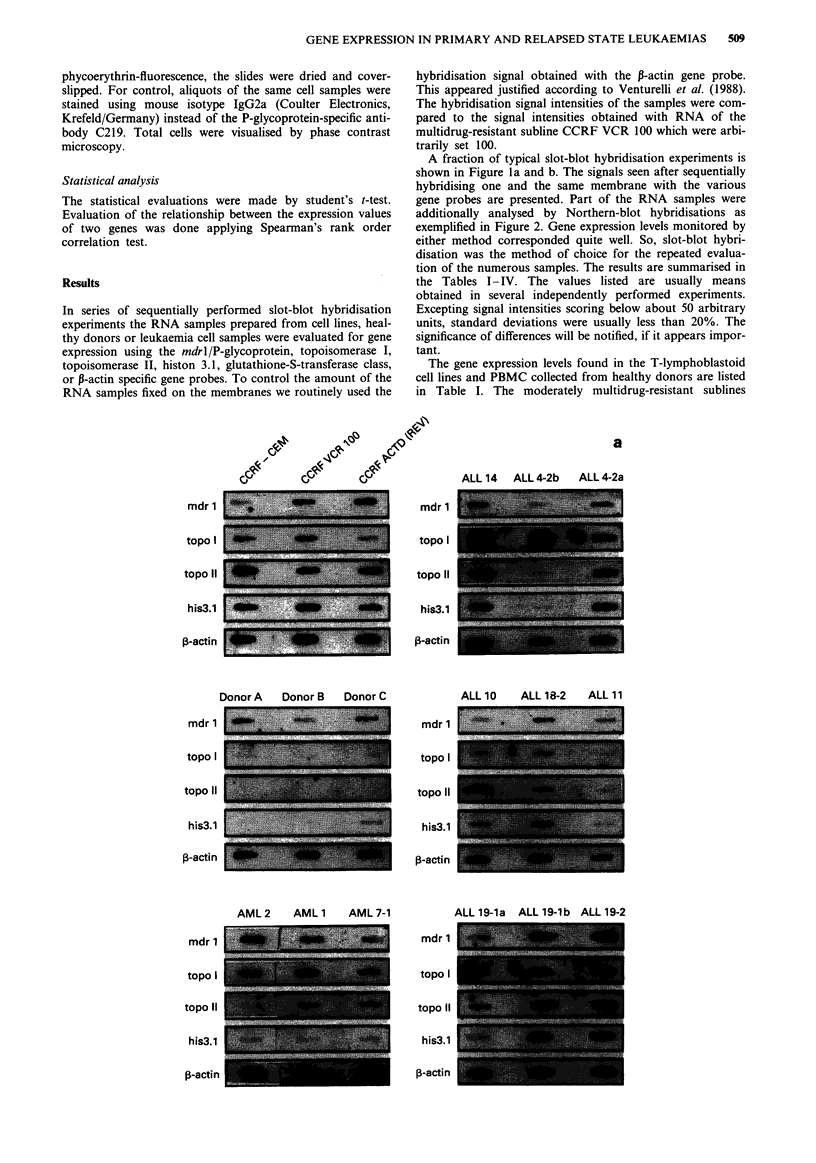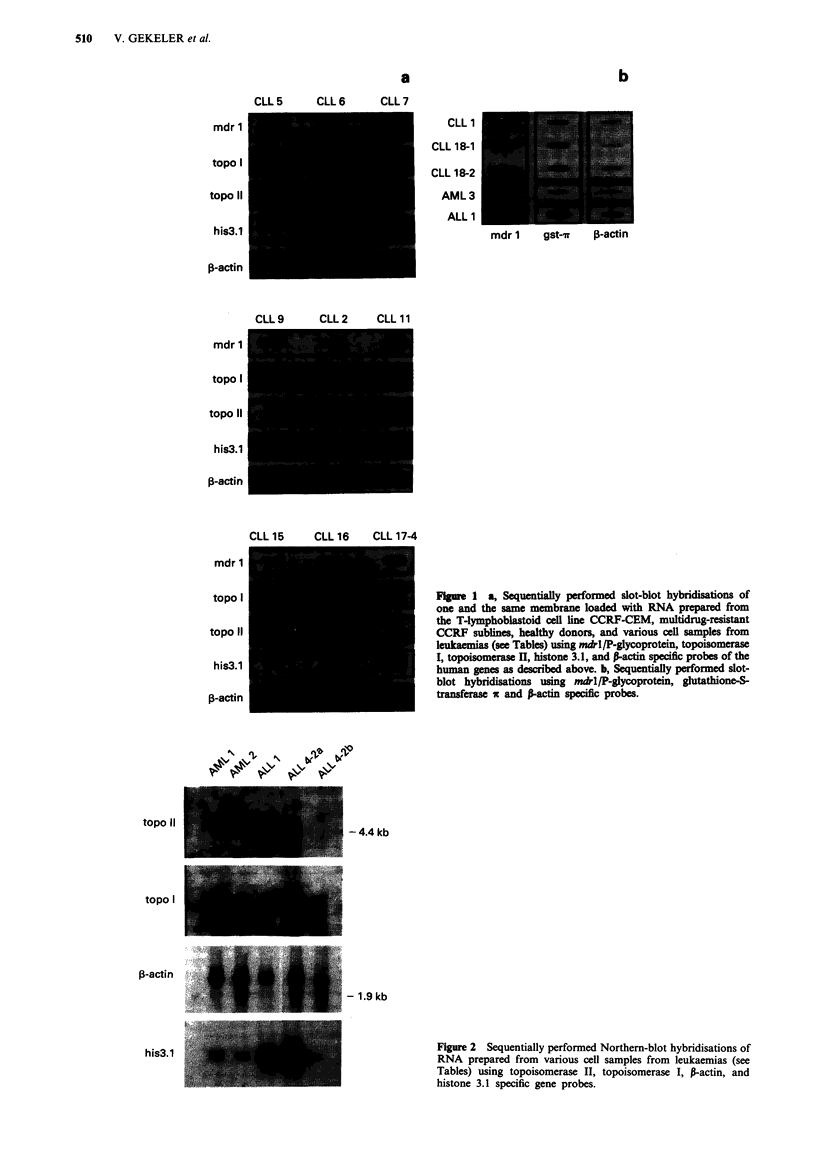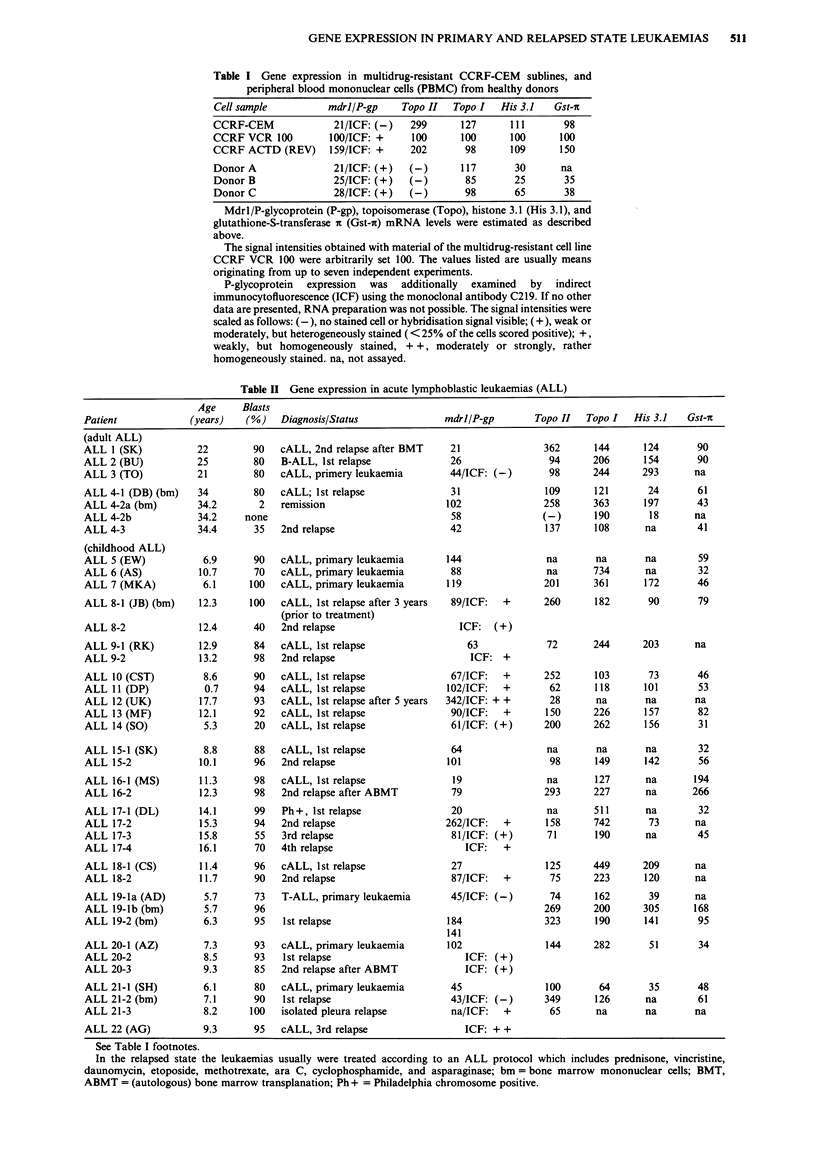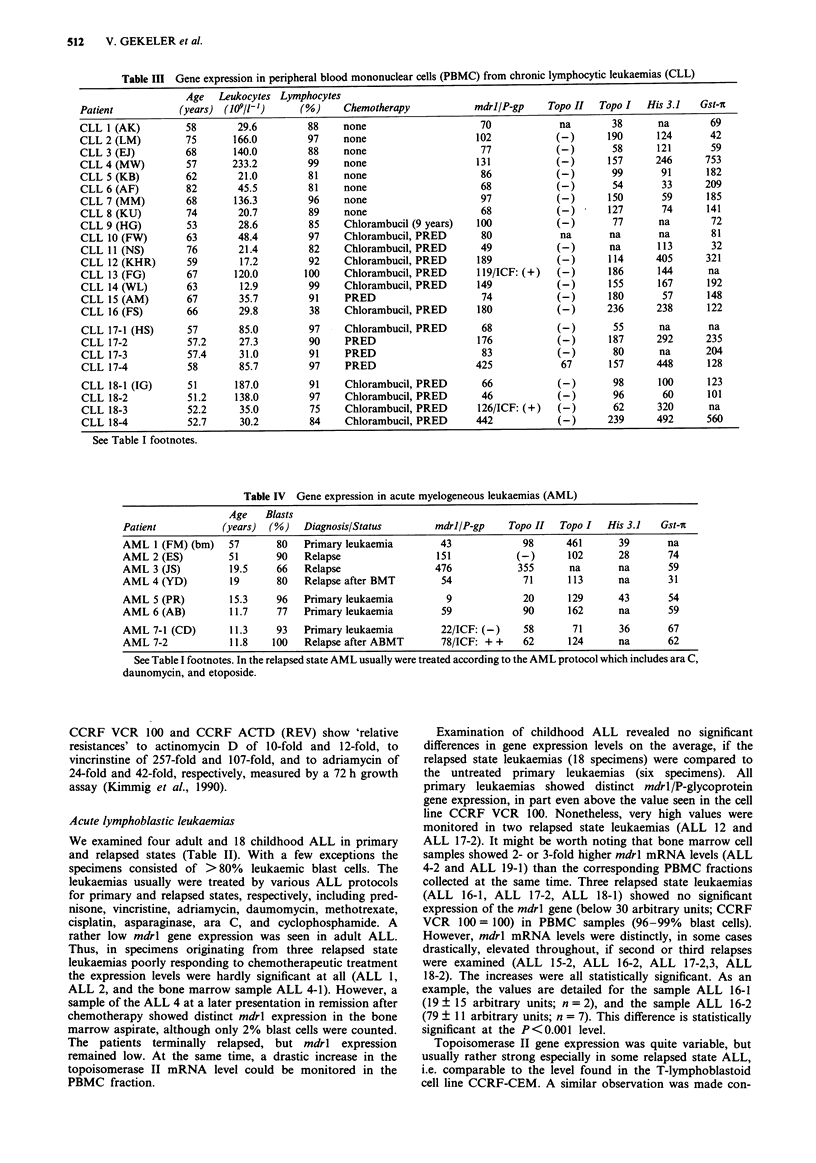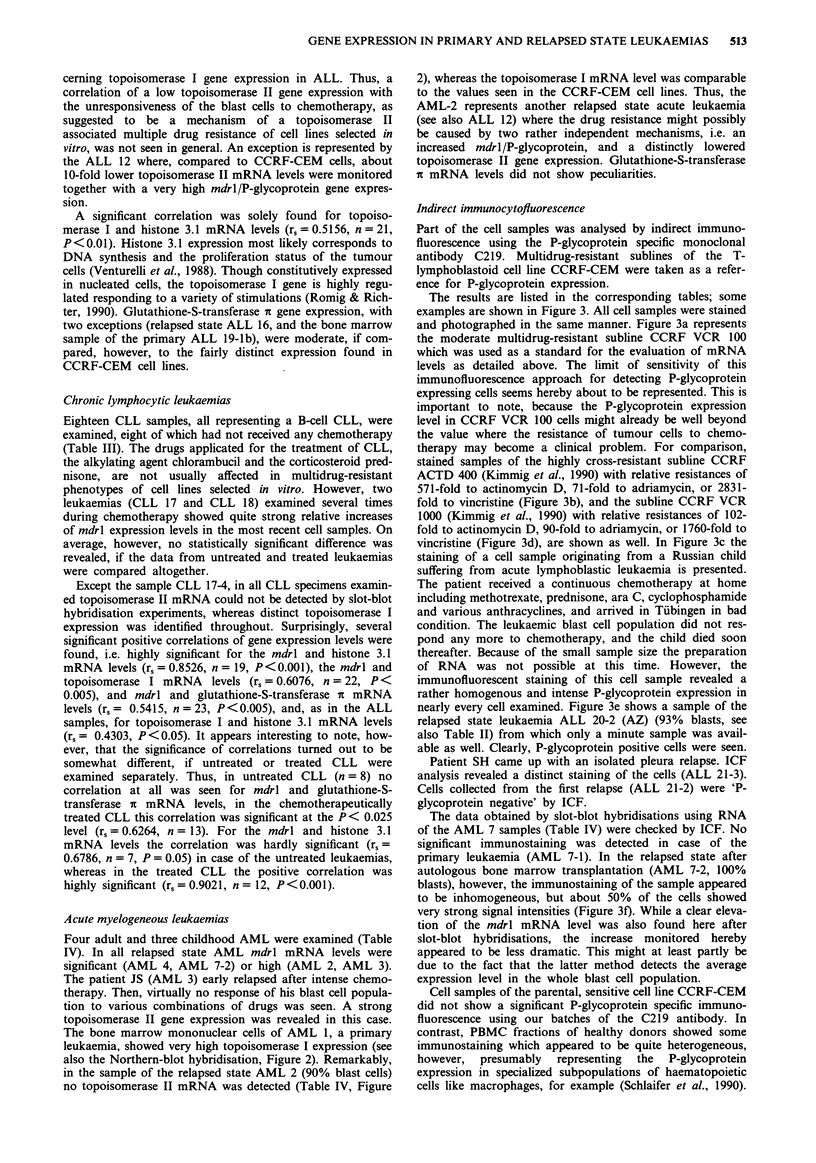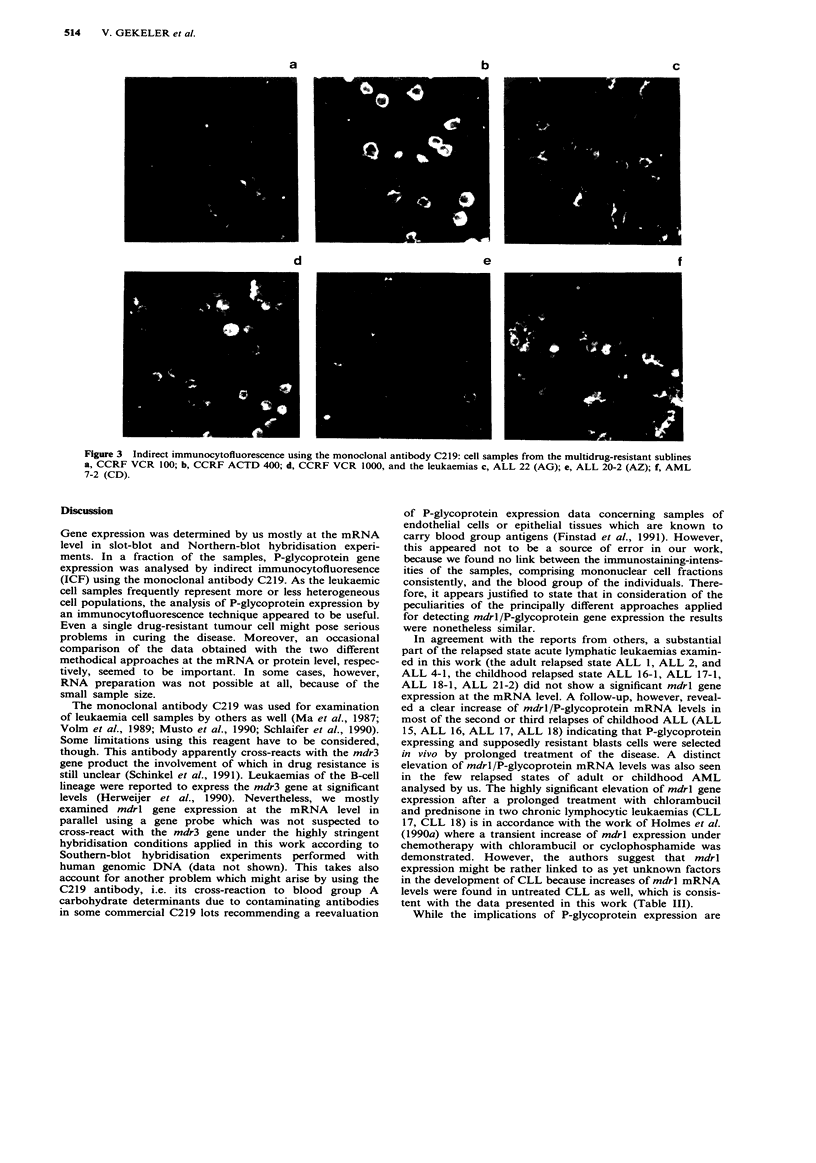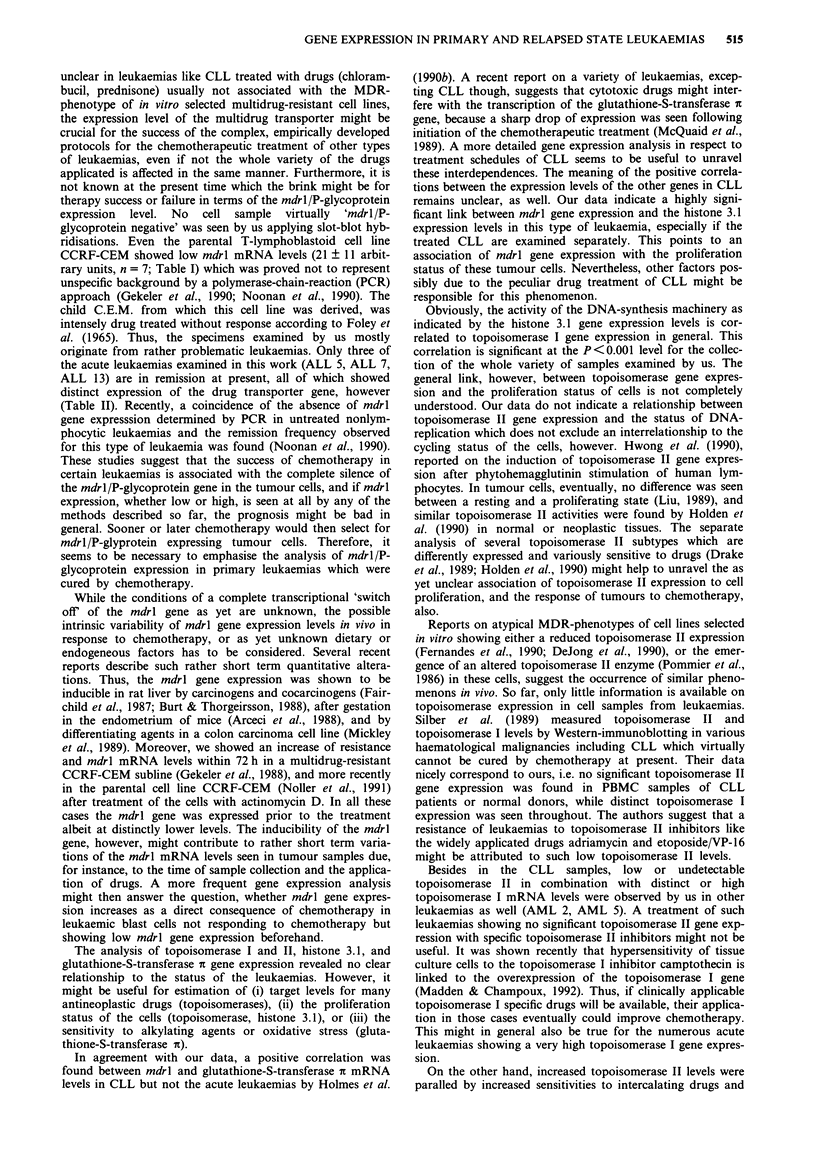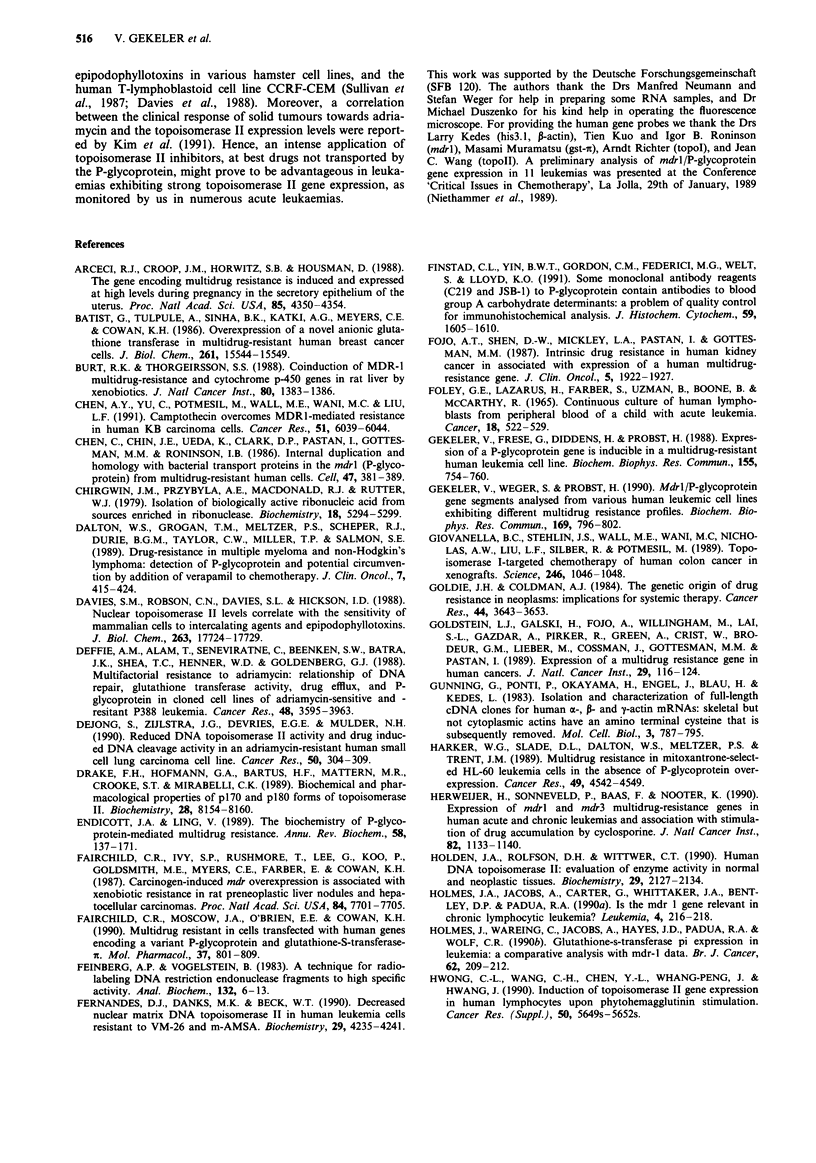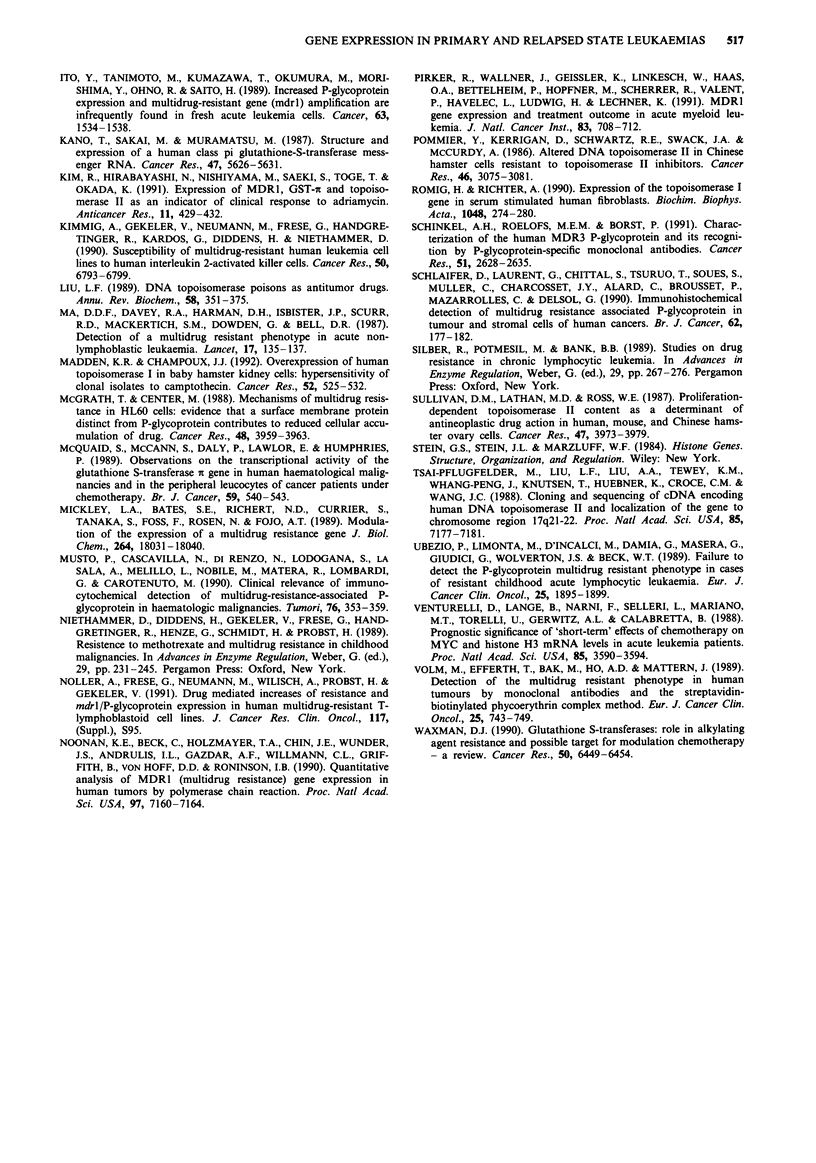# Mdr1/P-glycoprotein, topoisomerase, and glutathione-S-transferase pi gene expression in primary and relapsed state adult and childhood leukaemias.

**DOI:** 10.1038/bjc.1992.304

**Published:** 1992-09

**Authors:** V. Gekeler, G. Frese, A. Noller, R. Handgretinger, A. Wilisch, H. Schmidt, C. P. Muller, R. Dopfer, T. Klingebiel, H. Diddens

**Affiliations:** Physiologisch-chemisches Institut, Universität Tübingen, Germany.

## Abstract

**Images:**


Br. J. Cancer (1992), 66, 507-517 © Macmillan Press Ltd., 1992

### *Mdr*1/P-glycoprotein, topoisomerase, and glutathione-S-transferase π gene expression in primary and relapsed state adult and childhood leukaemias

V. Gekeler[1], G. Frese[1], A. Noller[1], R. Handgretinger[2], A. Wilisch[1], H. Schmidt[3], C.P. Muller[3], R. Dopfer[2], T. Klingebiel[2], H. Diddens[4], H. Probst[1] & D. Niethammer[2]

[1]*Physiologisch-chemisches Institut der Universität Tübingen, D-7400;* [2]*Kinderklinik der Universität Tübingen;* [3]*Medizinische Klinik der Universität Tübingen;* [4]*Medizinisches Laserzentrum, D-2400 Lübeck, Germany.*

**Summary** In a variety of adult and childhood leukaemia cell samples collected at different states of the disease, we analysed in a series of sequentially performed slot-blot or Northern-blot hybridisation experiments the expression of genes possibly involved in multiple drug resistance (MDR) (*mdr*1/P-glycoprotein, DNA topoisomerase II, glutathione-S-transferase π), and the expression of the DNA topoisomerase I and histone 3.1 genes. Occasionally, P-glycoprotein gene expression was additionally examined by indirect immunocytofluorescence using the monoclonal antibody C219. No significant difference in *mdr*1/P-glycoprotein mRNA levels between primary and relapsed state acute lymphocytic leukaemias (ALL) was seen on average. Second or third relapses, however, showed a distinct tendency to an elevated expression of this multidrug transporter gene (up to 10-fold) in part well beyond the value seen in the moderately cross-resistant T-lymphoblastoid CCRF-CEM subline CCRF VCR 100. Increased *mdr*1/P-glycoprotein mRNA levels were also found in relapsed state acute myelogeneous leukaemias (AML), and in chronic lymphocytic leukaemias (CLL) treated with chlorambucil and/or prednisone for several years. Topoisomerase I and topoisomerase II mRNA levels were found to be very variable. Whereas in all but one case of CLL topoisomerase II mRNA was not detected by slot-blot hybridisations, strong topoisomerase I and topoisomerase II gene expression levels, frequently exceeding the levels monitored in the CCRF-CEM cell line, were seen in many cell samples of acute leukaemia. If topoisomerase II mRNA was undetectable, expression of topoisomerase I was clearly visible throughout. These observations might be valuable considering the possible treatment with specific topoisomerase I or topoisomerase II inhibitors. Significant positive correlations were found (i) for topoisomerase I and histone 3.1 gene expression levels in general ($P < 0.001$), and (ii) in the CLL samples additionally for the expression levels of the *mdr*1 gene, and the histone 3.1, topoisomerase I, and glutathione-S-transferase π genes, respectively.

Failure of chemotherapy during treatment of leukaemia is supposedly caused by the resistance of the tumour cells to antineoplastic drugs (Goldie & Coldman, 1984). Multidrug resistant phenotypes of cultured cells selected *in vitro* have been intensely studied in recent years. Two different mechanisms confering resistance on these cells to a wide variety of structurally unrelated cytotoxic agents have been identified so far at the molecular level, (i) the enhanced expression of the *mdr*1 gene coding for an ATP-dependent, transmembrane drug efflux pump called P-glycoprotein, and (ii) an altered activity of the DNA topoisomerase II, a nuclear enzyme possessing DNA double-strand passing activity by an ATP-dependent cleaving and rejoining process. 'Classical' multiple drug resistance (MDR) of cell lines selected *in vitro* is basically mediated by the P-glycoprotein (for review see Endicott & Ling, 1989). The so-called 'atypical' MDR (at-MDR) where the numerous topoisomerase II inhibitors are affected could be associated with a quantitatively or qualitatively altered activity of topoisomerase II (Pommier *et al.*, 1986; Fernandes *et al.*, 1990; DeJong *et al.*, 1990). Other investigations, however, point to further as yet unrecognised mechanisms or a multifactorial emergence of MDR of *in vitro* selected cell lines (McGrath & Center, 1988; Deffie *et al.*, 1988; Harker *et al.*, 1989).

Clinical success of cancer therapy is strongly linked to the type and status of the tumour. This is exemplified by haematological malignancies. In general, childhood acute lymphoblastic leukaemias (ALL) usually respond well to complex protocols of intermittent chemotherapy applying combinations of various antineoplastic drugs, whereas the prognosis for adult ALL is worse. Showing lower curability than ALL the childhood acute myelogeneous leukaemias (AML) are still far better responding than adult AML. On the other hand, chronic lymphocytic leukaemias (CLL) which are only seen in adults usually receive either no chemotherapy at all, or if the disease is in progress, a combination of an alkylating agent and prednisone. A cure of CLL is usually not possible. In the case of the most prevalent childhood leukaemia, the acute lymphoblastic leukaemia, currently only about 20% relapse. Without bone marrow transplantation, the prognosis of relapsed leukaemia at present is bad, which actually might be due to the emergence of tumour cells less sensitive to antineoplastic agents. The molecular mechanisms of this clinically observed refractoriness of tumours to chemotherapy are still little understood. Several observations, however, point to the involvement of the P-glycoprotein, as (i) the unresponsiveness of tumours derived from tissues with an intrinsically high P-glycoprotein expression (Fojo *et al.*, 1987), (ii) the emergence of P-glycoprotein expression in specimens of relapsed state malignancies after chemotherapy (Ma *et al.*, 1987; Goldstein *et al.*, 1989; Volm *et al.*, 1989; Musto *et al.*, 1990; Pirker *et al.*, 1991), and (iii) reports on the successful treatment of drug-resistant tumours by including the calcium channel blocker and P-glycoprotein binding drug verapamil in chemotherapy protocols (Dalton *et al.*, 1989). However, other studies do not support the view of a frequently occurring link between elevated P-glycoprotein levels and therapy failures of leukaemias (Ito *et al.*, 1989; Ubezio *et al.*, 1989). Moreover, many clinically used antineoplastic drugs like ara C, cisplatin, or most alkylating agents do not even belong to the group of agents involved in the MDR-phenotype of cells selected *in vitro*. Hence, different mechanisms conferring drug resistance on tumour cells *in vivo* have to be considered.

Many antineoplastic drugs used clinically are inhibitors of the topoisomerase II and substrates for the P-glycoprotein mediated efflux as well, for instance, anthracyclines like adriamycin, or epipodophyllotoxins like teniposide. It has been suggested that the clinical response to epipodophyllotoxins could be dependent on a cell's topoisomerase II level. These drugs apparently stabilise an intermediate in the topoisomerase II catalysed reaction with the consequence of DNA

Correspondence: V. Gekeler, Physiologisch-chemisches Institut der Universität Tübingen, Hoppe-Seyler Strasse 4, 7400 Tübingen, Germany.
Received 19 November 1992; and in revised form 23 April 1992.

damage which might be lethal to the cell. Thus, reduced levels of the topoisomerase II could confer resistance to drugs targeted at the topoisomerase II by giving better chances to the cell's DNA repair systems (Holden et al., 1990). On the other hand, high topoisomerase II levels vice versa might be indicative for a good response of tumours to these kinds of drugs (Sullivan et al., 1987; Davies et al., 1988). Topoisomerase I is another nuclear enzyme involved in the regulation of DNA topology by relaxation of supercoils in an ATP-independent process (Liu, 1989). Camptothecins are specific inhibitors of topoisomerase I which are currently under investigation for use as antineoplastic drugs (Giovanella et al., 1989), and appear not to be affected in multidrug resistance (Chen et al., 1991).

The activity of the glutathione-S-transferase π was discussed as a further mechanism contributing to the MDR phenotype (Batist et al., 1986). It might, however, rather be involved in resistance of tumour cells to alkylating agents or oxidative stress (Fairchild et al., 1990; Waxman, 1990). The expression of the cell cycle regulated histone 3.1 gene represents an indicator of DNA synthesis activity (Stein et al., 1984; Venturelli et al., 1988) which is an important parameter, because DNA replication is one of the main targets of antineoplastic drugs.

Considering the possible complex genesis of a clinically observed refractoriness of tumours to treatment we analysed the expression of the *mdr*1/P-glycoprotein gene together with the variety of other genes in different types of leukaemias, at several times during progression of the disease. This might be helpful for the evaluation of drug resistance or an enhanced vulnerability of leukaemic cells to certain antineoplastic agents.

### Materials and methods

### *Materials*

Blotting membranes (Hybond $N^+$, Hybond N), labelling kits (Multiprime DNA Labelling System), [α-$^{32}$P]dCTP (specific activity > 3000 Ci mmol$^{-1}$; 1 Ci = 37 GBq) were obtained from Amersham (Braunschweig/Germany). All other chemicals, supplies and tissue culture media were of the purest grade and were purchased from commercial sources.

### *Cell lines and leukaemia cells samples*

The human T-lymphoblastoid cell line CCRF-CEM was obtained from the American Type Culture Collection, Rockville, MD/USA (ATCC CCL 119). The selection of multidrug-resistant CCRF-CEM sublines was published elsewhere (Gekeler et al., 1988; Niethammer et al., 1989). The cell lines used in this work were designated CCRF VCR 100, CCRF VCR 1000, and CCRF ACTD 400 according to the final vincristine or actinomycin D concentration (in ng ml$^{-1}$), respectively, used for selection and maintenance. A 'revertant' subline designated CCRF ACTD (REV) was maintained for more than a year without the drug. It showed a substantial decrease, though not a complete loss of resistance (Kimmig et al., 1990). Peripheral blood specimens or bone marrow aspirates were collected from normal donors and patients suffering from leukaemia in heparin without stabiliser. The mononuclear cells were concentrated by a standard Ficoll-Hypaque technique (Lymphoprep, Nycomed, Oslo/Norway), washed twice, frozen in the presence of 7% DMSO under controlled conditions, and stored in liquid nitrogen until used for analysis.

### *RNA isolation and analysis*

Total cellular RNA was extracted from the cell samples by lysis in guanidine thiocyanate, followed by centrifugation through cesium chloride (Chirgwin et al., 1979). The concentration of RNA in each sample was determined spectrophotometrically. Routinely, the quality of each RNA sample was monitored by ethidium bromide staining after electrophoresis in a 1% agarose/6% formaldehyde gel. For slot-blot hybridisations, 2.5 μg of each RNA sample were fixed onto Hybond $N^+$ membranes using the Minifold II slot blotting apparatus (Schleicher & Schüll, Dassel/Germany). For Northern-blot hybridisations 5 μg of total cellular RNA were electrophoresed in a 1% agarose/6% formaldehyde gel, and transferred by electroblotting onto Hybond $N^+$ membranes as recommended by the supplier (Amersham, Braunschweig/Germany). The RNA was fixed by UV-irradiation of the wet membranes using the Stratalinker 1800 (Stratagene, La Jolla/USA) as recommended by the supplier. Additionally, the membranes were baked thereafter at 80°C for 2 h. Thus, no significant loss of signal intensities were found after reprobing the filters up to six times.

As hybridisation probes we used the gel purified inserts of (i) the plasmid pcDR containing a 699 bp EcoRI cDNA fragment starting from position 1177 of the human P-glycoprotein mdr1 gene (Chen et al., 1986), (ii) the plasmid p3.2.4(M) containing a 2.2 kb cDNA EcoRI fragment of the human topoisomerase I gene (Romig & Richter, 1990), (iii) the plasmid phTOP2-1 containing a 2.4 kb cDNA EcoRI fragment of the human topoisomerase II gene (Tsai-Pflugfelder et al., 1988), (iv) the plasmid pLK288 containing a 1.7 kb EcoRI fragment of the human histone 3.1 gene, (v) the plasmid pGPi2 containing a 708 bp EcoRI cDNA fragment of the human glutathione-S-transferase class π gene (Kano et al., 1987), and (vi) the plasmid pHFβ-A1 containing a 2 kb BamHI fragment of the human β-actin cDNA (Gunning et al., 1983). The probes were labelled with [$^{32}$P]dCTP to a specific activity of $1-2 \times 10^9$ d.p.m. μg$^{-1}$ by 'oligolabelling' (Feinberg & Vogelstein, 1983), and used at a concentration of $1-2 \times 10^6$ d.p.m. ml$^{-1}$. The hybridisation procedure was performed as communicated earlier (Gekeler et al., 1988), besides 5% instead of 7% SDS (SDS = sodium dodecyl sulfate) were used. The filters were washed to a final stringency of 0.1 × SSC/0.1% SDS at 65°C (SSC = 0.15 M sodium chloride, 0.015 M sodium citrate), and autoradiographed with Hyperfilm MP (Amersham, Braunschweig/Germany) at − 80°C. For the quantitative evaluation of the autoradiographs by a Ultroscan XL 2222-20 laserdensitometer (Pharmacia-LKB, Freiburg/Germany) the films were exposed without intensifying screens. As size markers we used RNA-ladders purchased from Boehringer Mannheim/Germany or Gibco-BRL, Freiburg/Germany.

In pilot slot-blot hybridisation experiments we tested the performance of the *mdr*1 signal intensities using Hybond N or Hybond $N^+$ membranes (Amersham), respectively, in correlation to the amount of *mdr*1 mRNA in the sample. Therefore, 100 ng to 15 μg of total RNA obtained from the multidrug-resistant CCRF-CEM subline CCRF ACTD 400 were fixed onto the membranes as described. In the lower range (100 ng to 2.5 μg) *Escherichia coli* rRNA (Boehringer Mannheim) was added up to a final amount of 2.5 μg. Using the Hybond $N^+$ membrane stronger signal intensities together with a better approximation to a linear relationship between the amount of mRNA loaded and the signal intensity were obtained (data not shown).

### *Indirect immunocytofluorescence*

Cell suspensions of the CCRF-CEM cell lines or mononuclear cell fractions of the leukaemias were washed twice in ice-cold 0.9% sodium chloride, spotted onto gelatine coated slides and fixed in − 20°C cold acetone (fluorescence free, Merck, Darmstadt/Germany) and stored at − 80°C. According to Volm et al. (1989) we used the streptavidin-biotin-phycoerythrin method (Amersham). The fixed cells were incubated for 2 h with the monoclonal P-glycoprotein-specific antibody C219 at a concentration of 10 μg ml$^{-1}$ (Isotopen Diagnostik CIS, Dreieich/Germany). After washing, the cells were incubated with biotinylated sheep-anti-mouse second antibody, and then with the streptavidin-biotinylated-R-phycoerythrin-complex (Amersham). After addition of a stabiliser (Amersham) to prevent rapid fading of the phycoerythrin-fluorescence, the slides were dried and cover-slipped. For control, aliquots of the same cell samples were stained using mouse isotype IgG2a (Coulter Electronics, Krefeld/Germany) instead of the P-glycoprotein-specific anti-body C219. Total cells were visualised by phase contrast microscopy.

*Statistical analysis*

The statistical evaluations were made by student's *t*-test. Evaluation of the relationship between the expression values of two genes was done applying Spearman's rank order correlation test.

### Results

In series of sequentially performed slot-blot hybridisation experiments the RNA samples prepared from cell lines, healthy donors or leukaemia cell samples were evaluated for gene expression using the *mdr*1/P-glycoprotein, topoisomerase I, topoisomerase II, histon 3.1, glutathione-S-transferase class, or β-actin specific gene probes. To control the amount of the RNA samples fixed on the membranes we routinely used the hybridisation signal obtained with the β-actin gene probe. This appeared justified according to Venturelli *et al.* (1988). The hybridisation signal intensities of the samples were compared to the signal intensities obtained with RNA of the multidrug-resistant subline CCRF VCR 100 which were arbitrarily set 100.

A fraction of typical slot-blot hybridisation experiments is shown in Figure 1a and b. The signals seen after sequentially hybridising one and the same membrane with the various gene probes are presented. Part of the RNA samples were additionally analysed by Northern-blot hybridisations as exemplified in Figure 2. Gene expression levels monitored by either method corresponded quite well. So, slot-blot hybridisation was the method of choice for the repeated evaluation of the numerous samples. The results are summarised in the Tables I–IV. The values listed are usually means obtained in several independently performed experiments. Excepting signal intensities scoring below about 50 arbitrary units, standard deviations were usually less than 20%. The significance of differences will be notified, if it appears important.

The gene expression levels found in the T-lymphoblastoid cell lines and PBMC collected from healthy donors are listed in Table I. The moderately multidrug-resistant sublines

**Figure 1** **a**, Sequentially performed slot-blot hybridisations of one and the same membrane loaded with RNA prepared from the T-lymphoblastoid cell line CCRF-CEM, multidrug-resistant CCRF sublines, healthy donors, and various cell samples from leukaemias (see Tables) using *mdr*1/P-glycoprotein, topoisomerase I, topoisomerase II, histone 3.1, and β-actin specific probes of the human genes as described above. **b**, Sequentially performed slot-blot hybridisations using *mdr*1/P-glycoprotein, glutathione-S-transferase π and β-actin specific probes.

**Figure 2** Sequentially performed Northern-blot hybridisations of RNA prepared from various cell samples from leukaemias (see Tables) using topoisomerase II, topoisomerase I, β-actin, and histone 3.1 specific gene probes.

**Table I** Gene expression in multidrug-resistant CCRF-CEM sublines, and peripheral blood mononuclear cells (PBMC) from healthy donors

| *Cell sample* | *mdr1/P-gp* | *Topo II* | *Topo I* | *His 3.1* | *Gst-π* |
|---|---|---|---|---|---|
| CCRF-CEM | 21/ICF: (−) | 299 | 127 | 111 | 98 |
| CCRF VCR 100 | 100/ICF: + | 100 | 100 | 100 | 100 |
| CCRF ACTD (REV) | 159/ICF: + | 202 | 98 | 109 | 150 |
| Donor A | 21/ICF: (+) | (−) | 117 | 30 | na |
| Donor B | 25/ICF: (+) | (−) | 85 | 25 | 35 |
| Donor C | 28/ICF: (+) | (−) | 98 | 65 | 38 |

Mdr1/P-glycoprotein (P-gp), topoisomerase (Topo), histone 3.1 (His 3.1), and glutathione-S-transferase π (Gst-π) mRNA levels were estimated as described above.

The signal intensities obtained with material of the multidrug-resistant cell line CCRF VCR 100 were arbitrarily set 100. The values listed are usually means originating from up to seven independent experiments.

P-glycoprotein expression was additionally examined by indirect immunocytofluorescence (ICF) using the monoclonal antibody C219. If no other data are presented, RNA preparation was not possible. The signal intensities were scaled as follows: (−), no stained cell or hybridisation signal visible; (+), weak or moderately, but heterogeneously stained (<25% of the cells scored positive); +, weakly, but homogeneously stained, ++, moderately or strongly, rather homogeneously stained. na, not assayed.

**Table II** Gene expression in acute lymphoblastic leukaemias (ALL)

| *Patient* | *Age (years)* | *Blasts (%)* | *Diagnosis/Status* | *mdr1/P-gp* | *Topo II* | *Topo I* | *His 3.1* | *Gst-π* |
|---|---|---|---|---|---|---|---|---|
| (adult ALL) | | | | | | | | |
| ALL 1 (SK) | 22 | 90 | cALL, 2nd relapse after BMT | 21 | 362 | 144 | 124 | 90 |
| ALL 2 (BU) | 25 | 80 | B-ALL, 1st relapse | 26 | 94 | 206 | 154 | 90 |
| ALL 3 (TO) | 21 | 80 | cALL, primery leukaemia | 44/ICF: (−) | 98 | 244 | 293 | na |
| ALL 4-1 (DB) (bm) | 34 | 80 | cALL; 1st relapse | 31 | 109 | 121 | 24 | 61 |
| ALL 4-2a (bm) | 34.2 | 2 | remission | 102 | 258 | 363 | 197 | 43 |
| ALL 4-2b | 34.2 | none | | 58 | (−) | 190 | 18 | na |
| ALL 4-3 | 34.4 | 35 | 2nd relapse | 42 | 137 | 108 | na | 41 |
| (childhood ALL) | | | | | | | | |
| ALL 5 (EW) | 6.9 | 90 | cALL, primary leukaemia | 144 | na | na | na | 59 |
| ALL 6 (AS) | 10.7 | 70 | cALL, primary leukaemia | 88 | na | 734 | na | 32 |
| ALL 7 (MKA) | 6.1 | 100 | cALL, primary leukaemia | 119 | 201 | 361 | 172 | 46 |
| ALL 8-1 (JB) (bm) | 12.3 | 100 | cALL, 1st relapse after 3 years (prior to treatment) | 89/ICF: + | 260 | 182 | 90 | 79 |
| ALL 8-2 | 12.4 | 40 | 2nd relapse | ICF: (+) | | | | |
| ALL 9-1 (RK) | 12.9 | 84 | cALL, 1st relapse | 63 | 72 | 244 | 203 | na |
| ALL 9-2 | 13.2 | 98 | 2nd relapse | ICF: + | | | | |
| ALL 10 (CST) | 8.6 | 90 | cALL, 1st relapse | 67/ICF: + | 252 | 103 | 73 | 46 |
| ALL 11 (DP) | 0.7 | 94 | cALL, 1st relapse | 102/ICF: + | 62 | 118 | 101 | 53 |
| ALL 12 (UK) | 17.7 | 93 | cALL, 1st relapse after 5 years | 342/ICF: ++ | 28 | na | na | na |
| ALL 13 (MF) | 12.1 | 92 | cALL, 1st relapse | 90/ICF: + | 150 | 226 | 157 | 82 |
| ALL 14 (SO) | 5.3 | 20 | cALL, 1st relapse | 61/ICF: (+) | 200 | 262 | 156 | 31 |
| ALL 15-1 (SK) | 8.8 | 88 | cALL, 1st relapse | 64 | na | na | na | 32 |
| ALL 15-2 | 10.1 | 96 | 2nd relapse | 101 | 98 | 149 | 142 | 56 |
| ALL 16-1 (MS) | 11.3 | 98 | cALL, 1st relapse | 19 | na | 127 | na | 194 |
| ALL 16-2 | 12.3 | 98 | 2nd relapse after ABMT | 79 | 293 | 227 | na | 266 |
| ALL 17-1 (DL) | 14.1 | 99 | Ph+, 1st relapse | 20 | na | 511 | na | 32 |
| ALL 17-2 | 15.3 | 94 | 2nd relapse | 262/ICF: + | 158 | 742 | 73 | na |
| ALL 17-3 | 15.8 | 55 | 3rd relapse | 81/ICF: (+) | 71 | 190 | na | 45 |
| ALL 17-4 | 16.1 | 70 | 4th relapse | ICF: + | | | | |
| ALL 18-1 (CS) | 11.4 | 96 | cALL, 1st relapse | 27 | 125 | 449 | 209 | na |
| ALL 18-2 | 11.7 | 90 | 2nd relapse | 87/ICF: + | 75 | 223 | 120 | na |
| ALL 19-1a (AD) | 5.7 | 73 | T-ALL, primary leukaemia | 45/ICF: (−) | 74 | 162 | 39 | na |
| ALL 19-1b (bm) | 5.7 | 96 | | | 269 | 200 | 305 | 168 |
| ALL 19-2 (bm) | 6.3 | 95 | 1st relapse | 184<br>141 | 323 | 190 | 141 | 95 |
| ALL 20-1 (AZ) | 7.3 | 93 | cALL, primary leukaemia | 102 | 144 | 282 | 51 | 34 |
| ALL 20-2 | 8.5 | 93 | 1st relapse | ICF: (+) | | | | |
| ALL 20-3 | 9.3 | 85 | 2nd relapse after ABMT | ICF: (+) | | | | |
| ALL 21-1 (SH) | 6.1 | 80 | cALL, primary leukaemia | 45 | 100 | 64 | 35 | 48 |
| ALL 21-2 (bm) | 7.1 | 90 | 1st relapse | 43/ICF: (−) | 349 | 126 | na | 61 |
| ALL 21-3 | 8.2 | 100 | isolated pleura relapse | na/ICF: + | 65 | na | na | na |
| ALL 22 (AG) | 9.3 | 95 | cALL, 3rd relapse | ICF: ++ | | | | |

See Table I footnotes.

In the relapsed state the leukaemias usually were treated according to an ALL protocol which includes prednisone, vincristine, daunomycin, etoposide, methotrexate, ara C, cyclophosphamide, and asparaginase; bm = bone marrow mononuclear cells; BMT, ABMT = (autologous) bone marrow transplanation; Ph+ = Philadelphia chromosome positive.

**Table III** Gene expression in peripheral blood mononuclear cells (PBMC) from chronic lymphocytic leukaemias (CLL)

| *Patient* | *Age (years)* | *Leukocytes ($10^9 l^{-1}$)* | *Lymphocytes (%)* | *Chemotherapy* | *mdr1/P-gp* | *Topo II* | *Topo I* | *His 3.1* | *Gst-π* |
|---|---|---|---|---|---|---|---|---|---|
| CLL 1 (AK) | 58 | 29.6 | 88 | none | 70 | na | 38 | na | 69 |
| CLL 2 (LM) | 75 | 166.0 | 97 | none | 102 | (−) | 190 | 124 | 42 |
| CLL 3 (EJ) | 68 | 140.0 | 88 | none | 77 | (−) | 58 | 121 | 59 |
| CLL 4 (MW) | 57 | 233.2 | 99 | none | 131 | (−) | 157 | 246 | 753 |
| CLL 5 (KB) | 62 | 21.0 | 81 | none | 86 | (−) | 99 | 91 | 182 |
| CLL 6 (AF) | 82 | 45.5 | 81 | none | 68 | (−) | 54 | 33 | 209 |
| CLL 7 (MM) | 68 | 136.3 | 96 | none | 97 | (−) | 150 | 59 | 185 |
| CLL 8 (KU) | 74 | 20.7 | 89 | none | 68 | (−) | 127 | 74 | 141 |
| CLL 9 (HG) | 53 | 28.6 | 85 | Chlorambucil (9 years) | 100 | (−) | 77 | na | 72 |
| CLL 10 (FW) | 63 | 48.4 | 97 | Chlorambucil, PRED | 80 | na | na | na | 81 |
| CLL 11 (NS) | 76 | 21.4 | 82 | Chlorambucil, PRED | 49 | (−) | na | 113 | 32 |
| CLL 12 (KHR) | 59 | 17.2 | 92 | Chlorambucil, PRED | 189 | (−) | 114 | 405 | 321 |
| CLL 13 (FG) | 67 | 120.0 | 100 | Chlorambucil, PRED | 119/ICF: (+) | (−) | 186 | 144 | na |
| CLL 14 (WL) | 63 | 12.9 | 99 | Chlorambucil, PRED | 149 | (−) | 155 | 167 | 192 |
| CLL 15 (AM) | 67 | 35.7 | 91 | PRED | 74 | (−) | 180 | 57 | 148 |
| CLL 16 (FS) | 66 | 29.8 | 38 | Chlorambucil, PRED | 180 | (−) | 236 | 238 | 122 |
| CLL 17-1 (HS) | 57 | 85.0 | 97 | Chlorambucil, PRED | 68 | (−) | 55 | na | na |
| CLL 17-2 | 57.2 | 27.3 | 90 | PRED | 176 | (−) | 187 | 292 | 235 |
| CLL 17-3 | 57.4 | 31.0 | 91 | PRED | 83 | (−) | 80 | na | 204 |
| CLL 17-4 | 58 | 85.7 | 97 | PRED | 425 | 67 | 157 | 448 | 128 |
| CLL 18-1 (IG) | 51 | 187.0 | 91 | Chlorambucil, PRED | 66 | (−) | 98 | 100 | 123 |
| CLL 18-2 | 51.2 | 138.0 | 97 | Chlorambucil, PRED | 46 | (−) | 96 | 60 | 101 |
| CLL 18-3 | 52.2 | 35.0 | 75 | Chlorambucil, PRED | 126/ICF: (+) | (−) | 62 | 320 | na |
| CLL 18-4 | 52.7 | 30.2 | 84 | Chlorambucil, PRED | 442 | (−) | 239 | 492 | 560 |

See Table I footnotes.

**Table IV** Gene expression in acute myelogeneous leukaemias (AML)

| *Patient* | *Age (years)* | *Blasts (%)* | *Diagnosis/Status* | *mdr1/P-gp* | *Topo II* | *Topo I* | *His 3.1* | *Gst-π* |
|---|---|---|---|---|---|---|---|---|
| AML 1 (FM) (bm) | 57 | 80 | Primary leukaemia | 43 | 98 | 461 | 39 | na |
| AML 2 (ES) | 51 | 90 | Relapse | 151 | (−) | 102 | 28 | 74 |
| AML 3 (JS) | 19.5 | 66 | Relapse | 476 | 355 | na | na | 59 |
| AML 4 (YD) | 19 | 80 | Relapse after BMT | 54 | 71 | 113 | na | 31 |
| AML 5 (PR) | 15.3 | 96 | Primary leukaemia | 9 | 20 | 129 | 43 | 54 |
| AML 6 (AB) | 11.7 | 77 | Primary leukaemia | 59 | 90 | 162 | na | 59 |
| AML 7-1 (CD) | 11.3 | 93 | Primary leukaemia | 22/ICF: (−) | 58 | 71 | 36 | 67 |
| AML 7-2 | 11.8 | 100 | Relapse after ABMT | 78/ICF: ++ | 62 | 124 | na | 62 |

See Table I footnotes. In the relapsed state AML usually were treated according to the AML protocol which includes ara C, daunomycin, and etoposide.

CCRF VCR 100 and CCRF ACTD (REV) show 'relative resistances' to actinomycin D of 10-fold and 12-fold, to vincrinstine of 257-fold and 107-fold, and to adriamycin of 24-fold and 42-fold, respectively, measured by a 72 h growth assay (Kimmig *et al.*, 1990).

*Acute lymphoblastic leukaemias*

We examined four adult and 18 childhood ALL in primary and relapsed states (Table II). With a few exceptions the specimens consisted of >80% leukaemic blast cells. The leukaemias usually were treated by various ALL protocols for primary and relapsed states, respectively, including prednisone, vincristine, adriamycin, daumomycin, methotrexate, cisplatin, asparaginase, ara C, and cyclophosphamide. A rather low *mdr*1 gene expression was seen in adult ALL. Thus, in specimens originating from three relapsed state leukaemias poorly responding to chemotherapeutic treatment the expression levels were hardly significant at all (ALL 1, ALL 2, and the bone marrow sample ALL 4-1). However, a sample of the ALL 4 at a later presentation in remission after chemotherapy showed distinct *mdr*1 expression in the bone marrow aspirate, although only 2% blast cells were counted. The patients terminally relapsed, but *mdr*1 expression remained low. At the same time, a drastic increase in the topoisomerase II mRNA level could be monitored in the PBMC fraction.

Examination of childhood ALL revealed no significant differences in gene expression levels on the average, if the relapsed state leukaemias (18 specimens) were compared to the untreated primary leukaemias (six specimens). All primary leukaemias showed distinct *mdr*1/P-glycoprotein gene expression, in part even above the value seen in the cell line CCRF VCR 100. Nonetheless, very high values were monitored in two relapsed state leukaemias (ALL 12 and ALL 17-2). It might be worth noting that bone marrow cell samples showed 2- or 3-fold higher *mdr*1 mRNA levels (ALL 4-2 and ALL 19-1) than the corresponding PBMC fractions collected at the same time. Three relapsed state leukaemias (ALL 16-1, ALL 17-2, ALL 18-1) showed no significant expression of the *mdr*1 gene (below 30 arbitrary units; CCRF VCR 100 = 100) in PBMC samples (96–99% blast cells). However, *mdr*1 mRNA levels were distinctly, in some cases drastically, elevated throughout, if second or third relapses were examined (ALL 15-2, ALL 16-2, ALL 17-2,3, ALL 18-2). The increases were all statistically significant. As an example, the values are detailed for the sample ALL 16-1 ($19 \pm 15$ arbitrary units; $n = 2$), and the sample ALL 16-2 ($79 \pm 11$ arbitrary units; $n = 7$). This difference is statistically significant at the $P < 0.001$ level.

Topoisomerase II gene expression was quite variable, but usually rather strong especially in some relapsed state ALL, i.e. comparable to the level found in the T-lymphoblastoid cell line CCRF-CEM. A similar observation was made concerning topoisomerase I gene expression in ALL. Thus, a correlation of a low topoisomerase II gene expression with the unresponsiveness of the blast cells to chemotherapy, as suggested to be a mechanism of a topoisomerase II associated multiple drug resistance of cell lines selected *in vitro*, was not seen in general. An exception is represented by the ALL 12 where, compared to CCRF-CEM cells, about 10-fold lower topoisomerase II mRNA levels were monitored together with a very high *mdr*1/P-glycoprotein gene expression.

A significant correlation was solely found for topoisomerase I and histone 3.1 mRNA levels ($r_s = 0.5156$, $n = 21$, $P < 0.01$). Histone 3.1 expression most likely corresponds to DNA synthesis and the proliferation status of the tumour cells (Venturelli *et al.*, 1988). Though constitutively expressed in nucleated cells, the topoisomerase I gene is highly regulated responding to a variety of stimulations (Romig & Richter, 1990). Glutathione-S-transferase π gene expression, with two exceptions (relapsed state ALL 16, and the bone marrow sample of the primary ALL 19-1b), were moderate, if compared, however, to the fairly distinct expression found in CCRF-CEM cell lines.

*Chronic lymphocytic leukaemias*

Eighteen CLL samples, all representing a B-cell CLL, were examined, eight of which had not received any chemotherapy (Table III). The drugs applicated for the treatment of CLL, the alkylating agent chlorambucil and the corticosteroid prednisone, are not usually affected in multidrug-resistant phenotypes of cell lines selected *in vitro*. However, two leukaemias (CLL 17 and CLL 18) examined several times during chemotherapy showed quite strong relative increases of *mdr*l expression levels in the most recent cell samples. On average, however, no statistically significant difference was revealed, if the data from untreated and treated leukaemias were compared altogether.

Except the sample CLL 17-4, in all CLL specimens examined topoisomerase II mRNA could not be detected by slot-blot hybridisation experiments, whereas distinct topoisomerase I expression was identified throughout. Surprisingly, several significant positive correlations of gene expression levels were found, i.e. highly significant for the *mdr*l and histone 3.1 mRNA levels ($r_s = 0.8526$, $n = 19$, $P < 0.001$), the *mdr*l and topoisomerase I mRNA levels ($r_s = 0.6076$, $n = 22$, $P < 0.005$), and *mdr*l and glutathione-S-transferase π mRNA levels ($r_s = 0.5415$, $n = 23$, $P < 0.005$), and, as in the ALL samples, for topoisomerase I and histone 3.1 mRNA levels ($r_s = 0.4303$, $P < 0.05$). It appears interesting to note, however, that the significance of correlations turned out to be somewhat different, if untreated or treated CLL were examined separately. Thus, in untreated CLL ($n = 8$) no correlation at all was seen for *mdr*l and glutathione-S-transferase π mRNA levels, in the chemotherapeutically treated CLL this correlation was significant at the $P < 0.025$ level ($r_s = 0.6264$, $n = 13$). For the *mdr*l and histone 3.1 mRNA levels the correlation was hardly significant ($r_s = 0.6786$, $n = 7$, $P = 0.05$) in case of the untreated leukaemias, whereas in the treated CLL the positive correlation was highly significant ($r_s = 0.9021$, $n = 12$, $P < 0.001$).

*Acute myelogeneous leukaemias*

Four adult and three childhood AML were examined (Table IV). In all relapsed state AML *mdr*l mRNA levels were significant (AML 4, AML 7-2) or high (AML 2, AML 3). The patient JS (AML 3) early relapsed after intense chemotherapy. Then, virtually no response of his blast cell population to various combinations of drugs was seen. A strong topoisomerase II gene expression was revealed in this case. The bone marrow mononuclear cells of AML 1, a primary leukaemia, showed very high topoisomerase I expression (see also the Northern-blot hybridisation, Figure 2). Remarkably, in the sample of the relapsed state AML 2 (90% blast cells) no topoisomerase II mRNA was detected (Table IV, Figure 2), whereas the topoisomerase I mRNA level was comparable to the values seen in the CCRF-CEM cell lines. Thus, the AML-2 represents another relapsed state acute leukaemia (see also ALL 12) where the drug resistance might possibly be caused by two rather independent mechanisms, i.e. an increased *mdr*1/P-glycoprotein, and a distinctly lowered topoisomerase II gene expression. Glutathione-S-transferase π mRNA levels did not show peculiarities.

*Indirect immunocytofluorescence*

Part of the cell samples was analysed by indirect immunofluorescence using the P-glycoprotein specific monoclonal antibody C219. Multidrug-resistant sublines of the T-lymphoblastoid cell line CCRF-CEM were taken as a reference for P-glycoprotein expression.

The results are listed in the corresponding tables; some examples are shown in Figure 3. All cell samples were stained and photographed in the same manner. Figure 3a represents the moderate multidrug-resistant subline CCRF VCR 100 which was used as a standard for the evaluation of mRNA levels as detailed above. The limit of sensitivity of this immunofluorescence approach for detecting P-glycoprotein expressing cells seems hereby about to be represented. This is important to note, because the P-glycoprotein expression level in CCRF VCR 100 cells might already be well beyond the value where the resistance of tumour cells to chemotherapy may become a clinical problem. For comparison, stained samples of the highly cross-resistant subline CCRF ACTD 400 (Kimmig *et al.*, 1990) with relative resistances of 571-fold to actinomycin D, 71-fold to adriamycin, or 2831-fold to vincristine (Figure 3b), and the subline CCRF VCR 1000 (Kimmig *et al.*, 1990) with relative resistances of 102-fold to actinomycin D, 90-fold to adriamycin, or 1760-fold to vincristine (Figure 3d), are shown as well. In Figure 3c the staining of a cell sample originating from a Russian child suffering from acute lymphoblastic leukaemia is presented. The patient received a continuous chemotherapy at home including methotrexate, prednisone, ara C, cyclophosphamide and various anthracyclines, and arrived in Tübingen in bad condition. The leukaemic blast cell population did not respond any more to chemotherapy, and the child died soon thereafter. Because of the small sample size the preparation of RNA was not possible at this time. However, the immunofluorescent staining of this cell sample revealed a rather homogenous and intense P-glycoprotein expression in nearly every cell examined. Figure 3e shows a sample of the relapsed state leukaemia ALL 20-2 (AZ) (93% blasts, see also Table II) from which only a minute sample was available as well. Clearly, P-glycoprotein positive cells were seen.

Patient SH came up with an isolated pleura relapse. ICF analysis revealed a distinct staining of the cells (ALL 21-3). Cells collected from the first relapse (ALL 21-2) were 'P-glycoprotein negative' by ICF.

The data obtained by slot-blot hybridisations using RNA of the AML 7 samples (Table IV) were checked by ICF. No significant immunostaining was detected in case of the primary leukaemia (AML 7-1). In the relapsed state after autologous bone marrow transplantation (AML 7-2, 100% blasts), however, the immunostaining of the sample appeared to be inhomogeneous, but about 50% of the cells showed very strong signal intensities (Figure 3f). While a clear elevation of the *mdr*l mRNA level was also found here after slot-blot hybridisations, the increase monitored hereby appeared to be less dramatic. This might at least partly be due to the fact that the latter method detects the average expression level in the whole blast cell population.

Cell samples of the parental, sensitive cell line CCRF-CEM did not show a significant P-glycoprotein specific immunofluorescence using our batches of the C219 antibody. In contrast, PBMC fractions of healthy donors showed some immunostaining which appeared to be quite heterogeneous, however, presumably representing the P-glycoprotein expression in specialized subpopulations of haematopoietic cells like macrophages, for example (Schlaifer *et al.*, 1990).

**Figure 3** Indirect immunocytofluorescence using the monoclonal antibody C219: cell samples from the multidrug-resistant sublines **a**, CCRF VCR 100; **b**, CCRF ACTD 400; **d**, CCRF VCR 1000, and the leukaemias **c**, ALL 22 (AG); **e**, ALL 20-2 (AZ); **f**, AML 7-2 (CD).

### Discussion

Gene expression was determined by us mostly at the mRNA level in slot-blot and Northern-blot hybridisation experiments. In a fraction of the samples, P-glycoprotein gene expression was analysed by indirect immunocytofluoresence (ICF) using the monoclonal antibody C219. As the leukaemic cell samples frequently represent more or less heterogeneous cell populations, the analysis of P-glycoprotein expression by an immunocytofluorescence technique appeared to be useful. Even a single drug-resistant tumour cell might pose serious problems in curing the disease. Moreover, an occasional comparison of the data obtained with the two different methodical approaches at the mRNA or protein level, respectively, seemed to be important. In some cases, however, RNA preparation was not possible at all, because of the small sample size.

The monoclonal antibody C219 was used for examination of leukaemia cell samples by others as well (Ma *et al.*, 1987; Volm *et al.*, 1989; Musto *et al.*, 1990; Schlaifer *et al.*, 1990). Some limitations using this reagent have to be considered, though. This antibody apparently cross-reacts with the *mdr*3 gene product the involvement of which in drug resistance is still unclear (Schinkel *et al.*, 1991). Leukaemias of the B-cell lineage were reported to express the *mdr*3 gene at significant levels (Herweijer *et al.*, 1990). Nevertheless, we mostly examined *mdr*l gene expression at the mRNA level in parallel using a gene probe which was not suspected to cross-react with the *mdr*3 gene under the highly stringent hybridisation conditions applied in this work according to Southern-blot hybridisation experiments performed with human genomic DNA (data not shown). This takes also account for another problem which might arise by using the C219 antibody, i.e. its cross-reaction to blood group A carbohydrate determinants due to contaminating antibodies in some commercial C219 lots recommending a reevaluation of P-glycoprotein expression data concerning samples of endothelial cells or epithelial tissues which are known to carry blood group antigens (Finstad *et al.*, 1991). However, this appeared not to be a source of error in our work, because we found no link between the immunostaining-intensities of the samples, comprising mononuclear cell fractions consistently, and the blood group of the individuals. Therefore, it appears justified to state that in consideration of the peculiarities of the principally different approaches applied for detecting *mdr*l/P-glycoprotein gene expression the results were nonetheless similar.

In agreement with the reports from others, a substantial part of the relapsed state acute lymphatic leukaemias examined in this work (the adult relapsed state ALL 1, ALL 2, and ALL 4-1, the childhood relapsed state ALL 16-1, ALL 17-1, ALL 18-1, ALL 21-2) did not show a significant *mdr*l gene expression at the mRNA level. A follow-up, however, revealed a clear increase of *mdr*l/P-glycoprotein mRNA levels in most of the second or third relapses of childhood ALL (ALL 15, ALL 16, ALL 17, ALL 18) indicating that P-glycoprotein expressing and supposedly resistant blasts cells were selected *in vivo* by prolonged treatment of the disease. A distinct elevation of *mdr*l/P-glycoprotein mRNA levels was also seen in the few relapsed states of adult or childhood AML analysed by us. The highly significant elevation of *mdr*l gene expression after a prolonged treatment with chlorambucil and prednisone in two chronic lymphocytic leukaemias (CLL 17, CLL 18) is in accordance with the work of Holmes *et al.* (1990*a*) where a transient increase of *mdr*l expression under chemotherapy with chlorambucil or cyclophosphamide was demonstrated. However, the authors suggest that *mdr*l expression might be rather linked to as yet unknown factors in the development of CLL because increases of *mdr*l mRNA levels were found in untreated CLL as well, which is consistent with the data presented in this work (Table III).

While the implications of P-glycoprotein expression are unclear in leukaemias like CLL treated with drugs (chlorambucil, prednisone) usually not associated with the MDR-phenotype of *in vitro* selected multidrug-resistant cell lines, the expression level of the multidrug transporter might be crucial for the success of the complex, empirically developed protocols for the chemotherapeutic treatment of other types of leukaemias, even if not the whole variety of the drugs applicated is affected in the same manner. Furthermore, it is not known at the present time which the brink might be for therapy success or failure in terms of the *mdr*1/P-glycoprotein expression level. No cell sample virtually '*mdr*1/P-glycoprotein negative' was seen by us applying slot-blot hybridisations. Even the parental T-lymphoblastoid cell line CCRF-CEM showed low *mdr*1 mRNA levels (21 ± 11 arbitrary units, $n = 7$; Table I) which was proved not to represent unspecific background by a polymerase-chain-reaction (PCR) approach (Gekeler *et al.*, 1990; Noonan *et al.*, 1990). The child C.E.M. from which this cell line was derived, was intensely drug treated without response according to Foley *et al.* (1965). Thus, the specimens examined by us mostly originate from rather problematic leukaemias. Only three of the acute leukaemias examined in this work (ALL 5, ALL 7, ALL 13) are in remission at present, all of which showed distinct expression of the drug transporter gene, however (Table II). Recently, a coincidence of the absence of *mdr*1 gene expresssion determined by PCR in untreated nonlymphocytic leukaemias and the remission frequency observed for this type of leukaemia was found (Noonan *et al.*, 1990). These studies suggest that the success of chemotherapy in certain leukaemias is associated with the complete silence of the *mdr*1/P-glycoprotein gene in the tumour cells, and if *mdr*1 expression, whether low or high, is seen at all by any of the methods described so far, the prognosis might be bad in general. Sooner or later chemotherapy would then select for *mdr*1/P-glyprotein expressing tumour cells. Therefore, it seems to be necessary to emphasise the analysis of *mdr*1/P-glycoprotein expression in primary leukaemias which were cured by chemotherapy.

While the conditions of a complete transcriptional 'switch off' of the *mdr*1 gene as yet are unknown, the possible intrinsic variability of *mdr*1 gene expression levels *in vivo* in response to chemotherapy, or as yet unknown dietary or endogeneous factors has to be considered. Several recent reports describe such rather short term quantitative alterations. Thus, the *mdr*1 gene expression was shown to be inducible in rat liver by carcinogens and cocarcinogens (Fairchild *et al.*, 1987; Burt & Thorgeirsson, 1988), after gestation in the endometrium of mice (Arceci *et al.*, 1988), and by differentiating agents in a colon carcinoma cell line (Mickley *et al.*, 1989). Moreover, we showed an increase of resistance and *mdr*1 mRNA levels within 72 h in a multidrug-resistant CCRF-CEM subline (Gekeler *et al.*, 1988), and more recently in the parental cell line CCRF-CEM (Noller *et al.*, 1991) after treatment of the cells with actinomycin D. In all these cases the *mdr*1 gene was expressed prior to the treatment albeit at distinctly lower levels. The inducibility of the *mdr*1 gene, however, might contribute to rather short term variations of the *mdr*1 mRNA levels seen in tumour samples due, for instance, to the time of sample collection and the application of drugs. A more frequent gene expression analysis might then answer the question, whether *mdr*1 gene expression increases as a direct consequence of chemotherapy in leukaemic blast cells not responding to chemotherapy but showing low *mdr*1 gene expression beforehand.

The analysis of topoisomerase I and II, histone 3.1, and glutathione-S-transferase $\pi$ gene expression revealed no clear relationship to the status of the leukaemias. However, it might be useful for estimation of (i) target levels for many antineoplastic drugs (topoisomerases), (ii) the proliferation status of the cells (topoisomerase, histone 3.1), or (iii) the sensitivity to alkylating agents or oxidative stress (glutathione-S-transferase $\pi$).

In agreement with our data, a positive correlation was found between *mdr*1 and glutathione-S-transferase $\pi$ mRNA levels in CLL but not the acute leukaemias by Holmes *et al.* (1990*b*). A recent report on a variety of leukaemias, excepting CLL though, suggests that cytotoxic drugs might interfere with the transcription of the glutathione-S-transferase $\pi$ gene, because a sharp drop of expression was seen following initiation of the chemotherapeutic treatment (McQuaid *et al.*, 1989). A more detailed gene expression analysis in respect to treatment schedules of CLL seems to be useful to unravel these interdependences. The meaning of the positive correlations between the expression levels of the other genes in CLL remains unclear, as well. Our data indicate a highly significant link between *mdr*1 gene expression and the histone 3.1 expression levels in this type of leukaemia, especially if the treated CLL are examined separately. This points to an association of *mdr*1 gene expression with the proliferation status of these tumour cells. Nevertheless, other factors possibly due to the peculiar drug treatment of CLL might be responsible for this phenomenon.

Obviously, the activity of the DNA-synthesis machinery as indicated by the histone 3.1 gene expression levels is correlated to topoisomerase I gene expression in general. This correlation is significant at the $P < 0.001$ level for the collection of the whole variety of samples examined by us. The general link, however, between topoisomerase gene expression and the proliferation status of cells is not completely understood. Our data do not indicate a relationship between topoisomerase II gene expression and the status of DNA-replication which does not exclude an interrelationship to the cycling status of the cells, however. Hwong *et al.* (1990), reported on the induction of topoisomerase II gene expression after phytohemagglutinin stimulation of human lymphocytes. In tumour cells, eventually, no difference was seen between a resting and a proliferating state (Liu, 1989), and similar topoisomerase II activities were found by Holden *et al.* (1990) in normal or neoplastic tissues. The separate analysis of several topoisomerase II subtypes which are differently expressed and variously sensitive to drugs (Drake *et al.*, 1989; Holden *et al.*, 1990) might help to unravel the as yet unclear association of topoisomerase II expression to cell proliferation, and the response of tumours to chemotherapy, also.

Reports on atypical MDR-phenotypes of cell lines selected *in vitro* showing either a reduced topoisomerase II expression (Fernandes *et al.*, 1990; DeJong *et al.*, 1990), or the emergence of an altered topoisomerase II enzyme (Pommier *et al.*, 1986) in these cells, suggest the occurrence of similar phenomenons *in vivo*. So far, only little information is available on topoisomerase expression in cell samples from leukaemias. Silber *et al.* (1989) measured topoisomerase II and topoisomerase I levels by Western-immunoblotting in various haematological malignancies including CLL which virtually cannot be cured by chemotherapy at present. Their data nicely correspond to ours, i.e. no significant topoisomerase II gene expression was found in PBMC samples of CLL patients or normal donors, while distinct topoisomerase I expression was seen throughout. The authors suggest that a resistance of leukaemias to topoisomerase II inhibitors like the widely applicated drugs adriamycin and etoposide/VP-16 might be attributed to such low topoisomerase II levels.

Besides in the CLL samples, low or undetectable topoisomerase II in combination with distinct or high topoisomerase I mRNA levels were observed by us in other leukaemias as well (AML 2, AML 5). A treatment of such leukaemias showing no significant topoisomerase II gene expression with specific topoisomerase II inhibitors might not be useful. It was shown recently that hypersensitivity of tissue culture cells to the topoisomerase I inhibitor camptothecin is linked to the overexpression of the topoisomerase I gene (Madden & Champoux, 1992). Thus, if clinically applicable topoisomerase I specific drugs will be available, their application in those cases eventually could improve chemotherapy. This might in general also be true for the numerous acute leukaemias showing a very high topoisomerase I gene expression.

On the other hand, increased topoisomerase II levels were paralled by increased sensitivities to intercalating drugs and epipodophyllotoxins in various hamster cell lines, and the human T-lymphoblastoid cell line CCRF-CEM (Sullivan et al., 1987; Davies et al., 1988). Moreover, a correlation between the clinical response of solid tumours towards adriamycin and the topoisomerase II expression levels were reported by Kim et al. (1991). Hence, an intense application of topoisomerase II inhibitors, at best drugs not transported by the P-glycoprotein, might prove to be advantageous in leukaemias exhibiting strong topoisomerase II gene expression, as monitored by us in numerous acute leukaemias.

This work was supported by the Deutsche Forschungsgemeinschaft (SFB 120). The authors thank the Drs Manfred Neumann and Stefan Weger for help in preparing some RNA samples, and Dr Michael Duszenko for his kind help in operating the fluorescence microscope. For providing the human gene probes we thank the Drs Larry Kedes (his3.1, β-actin), Tien Kuo and Igor B. Roninson (*mdr*1), Masami Muramatsu (gst-π), Arndt Richter (topoI), and Jean C. Wang (topoII). A preliminary analysis of *mdr*1/P-glycoprotein gene expression in 11 leukemias was presented at the Conference 'Critical Issues in Chemotherapy', La Jolla, 29th of January, 1989 (Niethammer et al., 1989).

### References

ARCECI, R.J., CROOP, J.M., HORWITZ, S.B. & HOUSMAN, D. (1988). The gene encoding multidrug resistance is induced and expressed at high levels during pregnancy in the secretory epithelium of the uterus. *Proc. Natl Acad. Sci. USA*, **85**, 4350–4354.

BATIST, G., TULPULE, A., SINHA, B.K., KATKI, A.G., MEYERS, C.E. & COWAN, K.H. (1986). Overexpression of a novel anionic glutathione transferase in multidrug-resistant human breast cancer cells. *J. Biol. Chem.*, **261**, 15544–15549.

BURT, R.K. & THORGEIRSSON, S.S. (1988). Coinduction of MDR-1 multidrug-resistance and cytochrome p-450 genes in rat liver by xenobiotics. *J. Natl Cancer Inst.*, **80**, 1383–1386.

CHEN, A.Y., YU, C., POTMESIL, M., WALL, M.E., WANI, M.C. & LIU, L.F. (1991). Camptothecin overcomes MDR1-mediated resistance in human KB carcinoma cells. *Cancer Res.*, **51**, 6039–6044.

CHEN, C., CHIN, J.E., UEDA, K., CLARK, D.P., PASTAN, I., GOTTESMAN, M.M. & RONINSON, I.B. (1986). Internal duplication and homology with bacterial transport proteins in the *mdr*1 (P-glycoprotein) from multidrug-resistant human cells. *Cell*, **47**, 381–389.

CHIRGWIN, J.M., PRZYBYLA, A.E., MACDONALD, R.J. & RUTTER, W.J. (1979). Isolation of biologically active ribonucleic acid from sources enriched in ribonuclease. *Biochemistry*, **18**, 5294–5299.

DALTON, W.S., GROGAN, T.M., MELTZER, P.S., SCHEPER, R.J., DURIE, B.G.M., TAYLOR, C.W., MILLER, T.P. & SALMON, S.E. (1989). Drug-resistance in multiple myeloma and non-Hodgkin's lymphoma: detection of P-glycoprotein and potential circumvention by addition of verapamil to chemotherapy. *J. Clin. Oncol.*, **7**, 415–424.

DAVIES, S.M., ROBSON, C.N., DAVIES, S.L. & HICKSON, I.D. (1988). Nuclear topoisomerase II levels correlate with the sensitivity of mammalian cells to intercalating agents and epipodophyllotoxins. *J. Biol. Chem.*, **263**, 17724–17729.

DEFFIE, A.M., ALAM, T., SENEVIRATNE, C., BEENKEN, S.W., BATRA, J.K., SHEA, T.C., HENNER, W.D. & GOLDENBERG, G.J. (1988). Multifactorial resistance to adriamycin: relationship of DNA repair, glutathione transferase activity, drug efflux, and P-glycoprotein in cloned cell lines of adriamycin-sensitive and -resitant P388 leukemia. *Cancer Res.*, **48**, 3595–3963.

DEJONG, S., ZIJLSTRA, J.G., DEVRIES, E.G.E. & MULDER, N.H. (1990). Reduced DNA topoisomerase II activity and drug induced DNA cleavage activity in an adriamycin-resistant human small cell lung carcinoma cell line. *Cancer Res.*, **50**, 304–309.

DRAKE, F.H., HOFMANN, G.A., BARTUS, H.F., MATTERN, M.R., CROOKE, S.T. & MIRABELLI, C.K. (1989). Biochemical and pharmacological properties of p170 and p180 forms of topoisomerase II. *Biochemistry*, **28**, 8154–8160.

ENDICOTT, J.A. & LING, V. (1989). The biochemistry of P-glycoprotein-mediated multidrug resistance. *Annu. Rev. Biochem.*, **58**, 137–171.

FAIRCHILD, C.R., IVY, S.P., RUSHMORE, T., LEE, G., KOO, P., GOLDSMITH, M.E., MYERS, C.E., FARBER, E. & COWAN, K.H. (1987). Carcinogen-induced *mdr* overexpression is associated with xenobiotic resistance in rat preneoplastic liver nodules and hepatocellular carcinomas. *Proc. Natl Acad. Sci. USA*, **84**, 7701–7705.

FAIRCHILD, C.R., MOSCOW, J.A., O'BRIEN, E.E. & COWAN, K.H. (1990). Multidrug resistant in cells transfected with human genes encoding a variant P-glycoprotein and glutathione-S-transferase-π. *Mol. Pharmacol.*, **37**, 801–809.

FEINBERG, A.P. & VOGELSTEIN, B. (1983). A technique for radiolabeling DNA restriction endonuclease fragments to high specific activity. *Anal. Biochem.*, **132**, 6–13.

FERNANDES, D.J., DANKS, M.K. & BECK, W.T. (1990). Decreased nuclear matrix DNA topoisomerase II in human leukemia cells resistant to VM-26 and m-AMSA. *Biochemistry*, **29**, 4235–4241.

FINSTAD, C.L., YIN, B.W.T., GORDON, C.M., FEDERICI, M.G., WELT, S. & LLOYD, K.O. (1991). Some monoclonal antibody reagents (C219 and JSB-1) to P-glycoprotein contain antibodies to blood group A carbohydrate determinants: a problem of quality control for immunohistochemical analysis. *J. Histochem. Cytochem.*, **59**, 1605–1610.

FOJO, A.T., SHEN, D.-W., MICKLEY, L.A., PASTAN, I. & GOTTESMAN, M.M. (1987). Intrinsic drug resistance in human kidney cancer in associated with expression of a human multidrug-resistance gene. *J. Clin. Oncol.*, **5**, 1922–1927.

FOLEY, G.E., LAZARUS, H., FARBER, S., UZMAN, B., BOONE, B. & MCCARTHY, R. (1965). Continuous culture of human lymphoblasts from peripheral blood of a child with acute leukemia. *Cancer*, **18**, 522–529.

GEKELER, V., FRESE, G., DIDDENS, H. & PROBST, H. (1988). Expression of a P-glycoprotein gene is inducible in a multidrug-resistant human leukemia cell line. *Biochem. Biophys. Res. Commun.*, **155**, 754–760.

GEKELER, V., WEGER, S. & PROBST, H. (1990). *Mdr*1/P-glycoprotein gene segments analysed from various human leukemic cell lines exhibiting different multidrug resistance profiles. *Biochem. Biophys. Res. Commun.*, **169**, 796–802.

GIOVANELLA, B.C., STEHLIN, J.S., WALL, M.E., WANI, M.C, NICHOLAS, A.W., LIU, L.F., SILBER, R. & POTMESIL, M. (1989). Topoisomerase I-targeted chemotherapy of human colon cancer in xenografts. *Science*, **246**, 1046–1048.

GOLDIE, J.H. & COLDMAN, A.J. (1984). The genetic origin of drug resistance in neoplasms: implications for systemic therapy. *Cancer Res.*, **44**, 3643–3653.

GOLDSTEIN, L.J., GALSKI, H., FOJO, A., WILLINGHAM, M., LAI, S.-L., GAZDAR, A., PIRKER, R., GREEN, A., CRIST, W., BRODEUR, G.M., LIEBER, M., COSSMAN, J., GOTTESMAN, M.M. & PASTAN, I. (1989). Expression of a multidrug resistance gene in human cancers. *J. Natl. Cancer Inst.*, **29**, 116–124.

GUNNING, G., PONTI, P., OKAYAMA, H., ENGEL, J., BLAU, H. & KEDES, L. (1983). Isolation and characterization of full-length cDNA clones for human α-, β- and γ-actin mRNAs: skeletal but not cytoplasmic actins have an amino terminal cysteine that is subsequently removed. *Mol. Cell. Biol.*, **3**, 787–795.

HARKER, W.G., SLADE, D.L., DALTON, W.S., MELTZER, P.S. & TRENT, J.M. (1989). Multidrug resistance in mitoxantrone-selected HL-60 leukemia cells in the absence of P-glycoprotein overexpression. *Cancer Res.*, **49**, 4542–4549.

HERWEIJER, H., SONNEVELD, P., BAAS, F. & NOOTER, K. (1990). Expression of *mdr*1 and *mdr*3 multidrug-resistance genes in human acute and chronic leukemias and association with stimulation of drug accumulation by cyclosporine. *J. Natl Cancer Inst.*, **82**, 1133–1140.

HOLDEN, J.A., ROLFSON, D.H. & WITTWER, C.T. (1990). Human DNA topoisomerase II: evaluation of enzyme activity in normal and neoplastic tissues. *Biochemistry*, **29**, 2127–2134.

HOLMES, J.A., JACOBS, A., CARTER, G., WHITTAKER, J.A., BENTLEY, D.P. & PADUA, R.A. (1990*a*). Is the mdr 1 gene relevant in chronic lymphocytic leukemia? *Leukemia*, **4**, 216–218.

HOLMES, J., WAREING, C., JACOBS, A., HAYES, J.D., PADUA, R.A. & WOLF, C.R. (1990*b*). Glutathione-s-transferase pi expression in leukemia: a comparative analysis with mdr-1 data. *Br. J. Cancer*, **62**, 209–212.

HWONG, C.-L., WANG, C.-H., CHEN, Y.-L., WHANG-PENG, J. & HWANG, J. (1990). Induction of topoisomerase II gene expression in human lymphocytes upon phytohemagglutinin stimulation. *Cancer Res. (Suppl.)*, **50**, 5649s–5652s.

ITO, Y., TANIMOTO, M., KUMAZAWA, T., OKUMURA, M., MORISHIMA, Y., OHNO, R. & SAITO, H. (1989). Increased P-glycoprotein expression and multidrug-resistant gene (mdr1) amplification are infrequently found in fresh acute leukemia cells. *Cancer*, **63**, 1534–1538.

KANO, T., SAKAI, M. & MURAMATSU, M. (1987). Structure and expression of a human class pi glutathione-S-transferase messenger RNA. *Cancer Res.*, **47**, 5626–5631.

KIM, R., HIRABAYASHI, N., NISHIYAMA, M., SAEKI, S., TOGE, T. & OKADA, K. (1991). Expression of MDR1, GST-π and topoisomerase II as an indicator of clinical response to adriamycin. *Anticancer Res.*, **11**, 429–432.

KIMMIG, A., GEKELER, V., NEUMANN, M., FRESE, G., HANDGRETINGER, R., KARDOS, G., DIDDENS, H. & NIETHAMMER, D. (1990). Susceptibility of multidrug-resistant human leukemia cell lines to human interleukin 2-activated killer cells. *Cancer Res.*, **50**, 6793–6799.

LIU, L.F. (1989). DNA topoisomerase poisons as antitumor drugs. *Annu. Rev. Biochem.*, **58**, 351–375.

MA, D.D.F., DAVEY, R.A., HARMAN, D.H., ISBISTER, J.P., SCURR, R.D., MACKERTICH, S.M., DOWDEN, G. & BELL, D.R. (1987). Detection of a multidrug resistant phenotype in acute non-lymphoblastic leukaemia. *Lancet*, **17**, 135–137.

MADDEN, K.R. & CHAMPOUX, J.J. (1992). Overexpression of human topoisomerase I in baby hamster kidney cells: hypersensitivity of clonal isolates to camptothecin. *Cancer Res.*, **52**, 525–532.

MCGRATH, T. & CENTER, M. (1988). Mechanisms of multidrug resistance in HL60 cells: evidence that a surface membrane protein distinct from P-glycoprotein contributes to reduced cellular accumulation of drug. *Cancer Res.*, **48**, 3959–3963.

MCQUAID, S., MCCANN, S., DALY, P., LAWLOR, E. & HUMPHRIES, P. (1989). Observations on the transcriptional activity of the glutathione S-transferase π gene in human haematological malignancies and in the peripheral leucocytes of cancer patients under chemotherapy. *Br. J. Cancer*, **59**, 540–543.

MICKLEY, L.A., BATES, S.E., RICHERT, N.D., CURRIER, S., TANAKA, S., FOSS, F., ROSEN, N. & FOJO, A.T. (1989). Modulation of the expression of a multidrug resistance gene *J. Biol. Chem.*, **264**, 18031–18040.

MUSTO, P., CASCAVILLA, N., DI RENZO, N., LODOGANA, S., LA SALA, A., MELILLO, L., NOBILE, M., MATERA, R., LOMBARDI, G. & CAROTENUTO, M. (1990). Clinical relevance of immunocytochemical detection of multidrug-resistance-associated P-glycoprotein in haematologic malignancies. *Tumori*, **76**, 353–359.

NIETHAMMER, D., DIDDENS, H., GEKELER, V., FRESE, G., HANDGRETINGER, R., HENZE, G., SCHMIDT, H. & PROBST, H. (1989). Resistence to methotrexate and multidrug resistance in childhood malignancies. In *Advances in Enzyme Regulation*, Weber, G. (ed.), 29, pp. 231–245. Pergamon Press: Oxford, New York.

NOLLER, A., FRESE, G., NEUMANN, M., WILISCH, A., PROBST, H. & GEKELER, V. (1991). Drug mediated increases of resistance and *mdr*1/P-glycoprotein expression in human multidrug-resistant T-lymphoblastoid cell lines. *J. Cancer Res. Clin. Oncol.*, **117**, (Suppl.), S95.

NOONAN, K.E., BECK, C., HOLZMAYER, T.A., CHIN, J.E., WUNDER, J.S., ANDRULIS, I.L., GAZDAR, A.F., WILLMANN, C.L., GRIFFITH, B., VON HOFF, D.D. & RONINSON, I.B. (1990). Quantitative analysis of MDR1 (multidrug resistance) gene expression in human tumors by polymerase chain reaction. *Proc. Natl Acad. Sci. USA*, **97**, 7160–7164.

PIRKER, R., WALLNER, J., GEISSLER, K., LINKESCH, W., HAAS, O.A., BETTELHEIM, P., HOPFNER, M., SCHERRER, R., VALENT, P., HAVELEC, L., LUDWIG, H. & LECHNER, K. (1991). MDR1 gene expression and treatment outcome in acute myeloid leukemia. *J. Natl. Cancer Inst.*, **83**, 708–712.

POMMIER, Y., KERRIGAN, D., SCHWARTZ, R.E., SWACK, J.A. & MCCURDY, A. (1986). Altered DNA topoisomerase II in Chinese hamster cells resistant to topoisomerase II inhibitors. *Cancer Res.*, **46**, 3075–3081.

ROMIG, H. & RICHTER, A. (1990). Expression of the topoisomerase I gene in serum stimulated human fibroblasts. *Biochim. Biophys. Acta.*, **1048**, 274–280.

SCHINKEL, A.H., ROELOFS, M.E.M. & BORST, P. (1991). Characterization of the human MDR3 P-glycoprotein and its recognition by P-glycoprotein-specific monoclonal antibodies. *Cancer Res.*, **51**, 2628–2635.

SCHLAIFER, D., LAURENT, G., CHITTAL, S., TSURUO, T., SOUES, S., MULLER, C., CHARCOSSET, J.Y., ALARD, C., BROUSSET, P., MAZARROLLES, C. & DELSOL, G. (1990). Immunohistochemical detection of multidrug resistance associated P-glycoprotein in tumour and stromal cells of human cancers. *Br. J. Cancer*, **62**, 177–182.

SILBER, R., POTMESIL, M. & BANK, B.B. (1989). Studies on drug resistance in chronic lymphocytic leukemia. In *Advances in Enzyme Regulation*, Weber, G. (ed.), 29, pp. 267–276. Pergamon Press: Oxford, New York.

SULLIVAN, D.M., LATHAN, M.D. & ROSS, W.E. (1987). Proliferation-dependent topoisomerase II content as a determinant of antineoplastic drug action in human, mouse, and Chinese hamster ovary cells. *Cancer Res.*, **47**, 3973–3979.

STEIN, G.S., STEIN, J.L. & MARZLUFF, W.F. (1984). *Histone Genes. Structure, Organization, and Regulation*. Wiley: New York.

TSAI-PFLUGFELDER, M., LIU, L.F., LIU, A.A., TEWEY, K.M., WHANG-PENG, J., KNUTSEN, T., HUEBNER, K., CROCE, C.M. & WANG, J.C. (1988). Cloning and sequencing of cDNA encoding human DNA topoisomerase II and localization of the gene to chromosome region 17q21-22. *Proc. Natl Acad. Sci. USA*, **85**, 7177–7181.

UBEZIO, P., LIMONTA, M., D'INCALCI, M., DAMIA, G., MASERA, G., GIUDICI, G., WOLVERTON, J.S. & BECK, W.T. (1989). Failure to detect the P-glycoprotein multidrug resistant phenotype in cases of resistant childhood acute lymphocytic leukaemia. *Eur. J. Cancer Clin. Oncol.*, **25**, 1895–1899.

VENTURELLI, D., LANGE, B., NARNI, F., SELLERI, L., MARIANO, M.T., TORELLI, U., GERWITZ, A.L. & CALABRETTA, B. (1988). Prognostic significance of 'short-term' effects of chemotherapy on MYC and histone H3 mRNA levels in acute leukemia patients. *Proc. Natl Acad. Sci. USA*, **85**, 3590–3594.

VOLM, M., EFFERTH, T., BAK, M., HO, A.D. & MATTERN, J. (1989). Detection of the multidrug resistant phenotype in human tumours by monoclonal antibodies and the streptavidin-biotinylated phycoerythrin complex method. *Eur. J. Cancer Clin. Oncol.*, **25**, 743–749.

WAXMAN, D.J. (1990). Glutathione S-transferases: role in alkylating agent resistance and possible target for modulation chemotherapy – a review. *Cancer Res.*, **50**, 6449–6454.